# The evaluation of tumorigenicity and characterization of colonies in a soft agar colony formation assay using polymerase chain reaction

**DOI:** 10.1038/s41598-023-32442-6

**Published:** 2023-04-03

**Authors:** Daichi Nakamura

**Affiliations:** 1 BoZo Research Center Inc., Tsukuba Research Institute, 8 Okubo, Tsukuba, Ibaraki 300-2611 Japan; 2 Graduate School of Life and Environmental Sciences, University of Tsukuba, 1-1-1 Tennodai, Tsukuba, Ibaraki 305-8572 Japan

**Keywords:** Biological techniques, Biotechnology, Cancer, Cell biology, Molecular biology, Stem cells, Biomarkers, Medical research, Oncology

## Abstract

In regenerative medicine, the tumorigenic potency of cells in cellular therapy products (CTPs) is a major concern for their application to patients. This study presents a method—the soft agar colony formation assay using polymerase chain reaction (PCR)—to evaluate tumorigenicity. MRC-5 cells, contaminated with HeLa cells, were cultured for up to 4 weeks in soft agar medium. Cell-proliferation-related mRNAs, Ki-67 and cyclin B, could be detected in 0.01% of HeLa cells after 5 days of culture, whereas cyclin-dependent kinase 1 (CDK1) could be detected after 2 weeks. On the other hand, CDK2, proliferating cell nuclear antigen (PCNA), and minichromosome maintenance protein 7 (MCM7) were not useful to detect HeLa cells even after 4 weeks of culture. The cancer stem cell (CSC) markers, aldehyde dehydrogenase 1 (ALDH1) and CD133 in 0.01% of HeLa cells, could be detected 2 and 4 weeks after culture, respectively. However, another CSC marker CD44 was not useful because its expression was also detected in MRC-5 cells alone. This study suggests that the application of the PCR method to the soft agar colony formation assay could evaluate not only the tumorigenic potency in the short-term but also characterize the colonies, eventually improving the safety of CTPs.

## Introduction

Cellular therapy products (CTPs), a recent development in regenerative medicine, are made from stem cells, like mesenchymal stem cells or induced pluripotent stem (iPS) cells. The tumorigenic potency of cells used in CTPs is a cause for great concern, for example, neural stem cells cultured in vitro^1^ or iPS cells transplanted in vivo^2^ have been reported to be tumorigenic. For gene therapy, 6 of 20 patients became leukemic after undergoing gene therapy for X-linked Severe Immune Deficiency (X-SCID) due to the promotion of oncogene expression^3^.

In vivo and in vitro tests have been developed to assess the tumorigenicity of CTPs. For an in vivo test, CTPs are injected into immune-deficient animals and observed for more than 16 weeks^2,4^. However, this study is costly and needs a long duration. As an in vitro test, the soft agar colony formation assay is widely used. This assay is based on the unique anchorage-independent cell growth exhibited by transformed cells. In normal cells, cell division is promoted by binding cells to the extracellular matrix, such as the bottom of the culture petri dish, to form a scaffold, but when they cannot bind to the extracellular matrix, that is, when there is no scaffold, cell division is arrested or apoptosis (called anoikis) occurs. However, in transformed cells, cell division continues in an anchorage-independent manner even in the absence of a scaffold. When cultured in soft agar, normal adherent cells either become quiescent or undergo anoikis due to detachment from the extracellular matrix, whereas tumorigenic cells continue to proliferate without anoikis and form a spheroid-like cell cluster, which is called colony. As a result, only malignant cells can proliferate and form colonies. Two methods have been introduced for detecting these colonies^5^: (i) DNA staining by an intercalator, which has a lower limit of detection (LLOD) of approximately 0.02% HeLa cells contaminated in mesenchymal cells and an assay duration of 20 days, (ii) Digital soft agar colony formation assay using cell image analysis, which has an LLOD of 0.00001% and an assay duration of 30 days.

Cancer stem cells (CSCs) comprise a minor population in the cancer tissue that is, however, responsible for chemotherapy resistance, cancer recurrence, metastasis, and unfavorable therapy outcomes^6^. Chen et al. succeeded in artificially generating CSCs from iPS cells using a conditioned medium^7^. This implies that CSCs can be accidentally generated from iPS or other stem cells by a simple condition such as a medium, and there is a risk of contamination of CTPs or its raw materials.

In this study, HeLa and MRC-5 cells were used as tumorigenic and normal cells, respectively. The human cervical cancer cell line HeLa was introduced as a positive reference for tumorigenicity test in the World Health Organization (WHO) technical report series no. 978^8^, and has been widely used in cancer research^4,5^. This cell line also contains CSCs^9–11^. On the other hand, MRC-5, a fetal lung, normal diploid fibroblast cell line, is widely used in research on biotherapeutics such as vaccines^8^. MRC-5 cells were used to establish some iPS cell strains deposited in the Japanese Collection of Research Bioresources (JCRB) Cell Bank^12^ and are expected to be raw materials for CTPs.

The expression of mRNAs is strictly regulated across different cellular activities, such as cell cycle, differentiation, etc., implying that a specific cellular state may be identified by a particular mRNA marker. For example, residual undifferentiated cells in CTPs have been detected using *LIN28* mRNA as a marker for undifferentiated cells^13,14^. During the different phases of cell proliferation, such as DNA replication, mitosis, or quiescence, many essential mRNAs are up- or down-regulated in a time-specific manner. Therefore, I hypothesized that tumorigenic cells proliferating in soft agar could be identified from their mRNAs in a much shorter time compared with DNA-staining or digital soft agar colony formation assays. Furthermore, this approach could characterize colonies grown in soft agar by other mRNA markers.

Because no report that assesses tumorigenicity using mRNAs exists, this study was conducted as the first trial using common cell lines, HeLa, and MRC-5 cell to demonstrate the mRNA analysis of colonies in soft agar via polymerase chain reaction (PCR). I could detect 0.01% HeLa cells and their CSC marker spiked in MRC-5 cells after 5 days and 2 weeks of cell culture, respectively.

## Results

### Ki-67, cyclin B, and CDK1 mRNA measurements detect 0.01% of HeLa cells in 5-day culture

**Figure 1:**
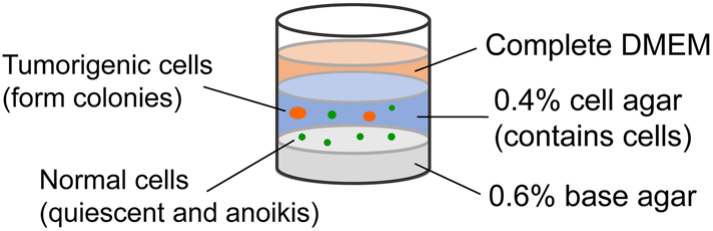
Schematic diagram of the soft agar colony formation assay. Cultured in a soft agar, normal cells (green color) become either quiescent or undergo anoikis, whereas malignant cells continue to proliferate and form colonies (orange color).

The soft agar colony formation assay was performed as shown in Fig. 1. Normal cells cultured in the soft agar cannot adhere to the scaffold, inducing cell cycle quiescence or anoikis, whereas malignant cells continue to proliferate and form cell colonies. There are two requirements for candidate markers for detecting tumorigenic cells: (i) marker expressions are very low or not detected during the G0 or quiescent phase, and (ii) expresses in the cell proliferation stages. Since the proteins Ki-67, cyclin-dependent kinase 1 (CDK1), cyclin B, and minichromosome maintenance protein 7 (MCM7) play major roles in DNA replication or mitosis, their mRNA expressions are expected to be useful markers to detect tumorigenic cells that can proliferate in the soft agar.

**Figure 2:**
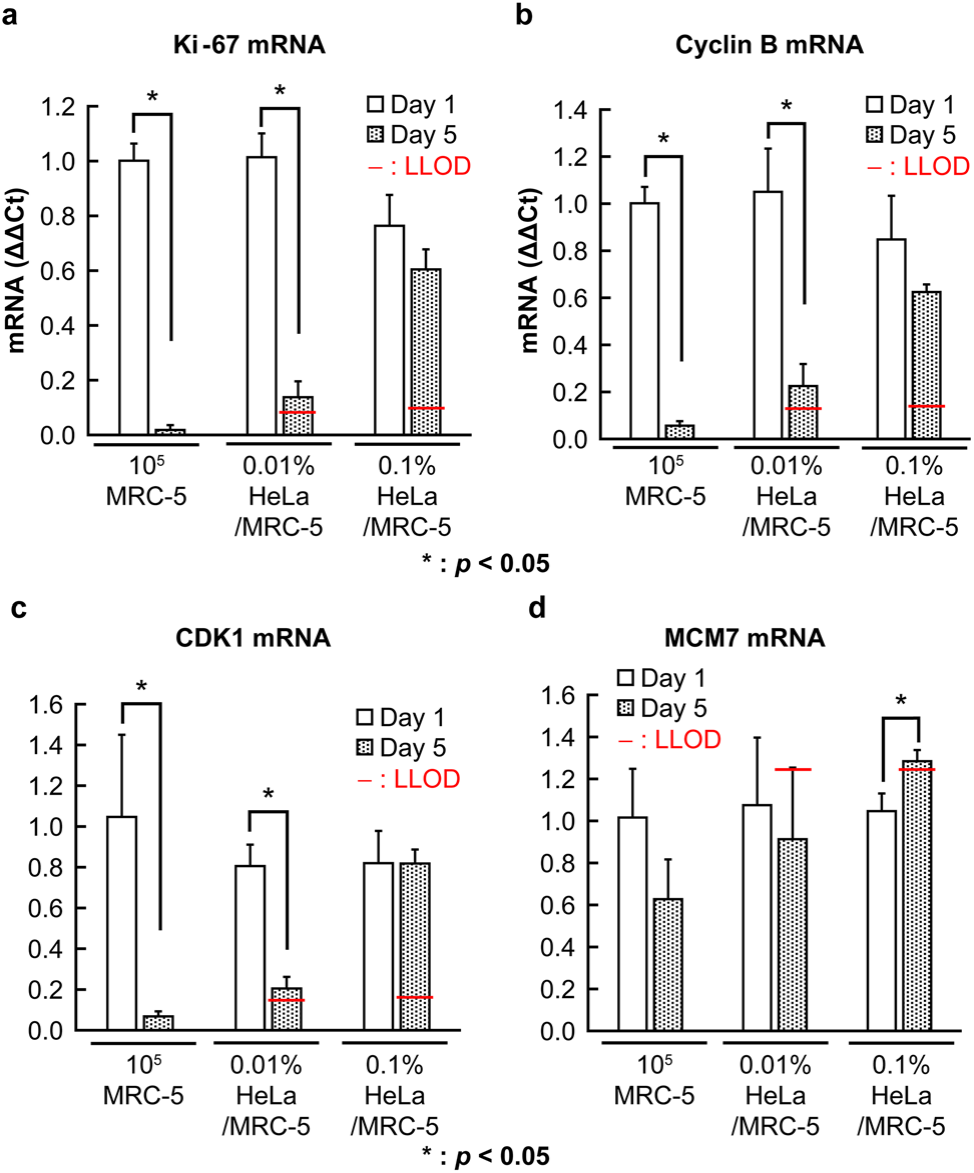
Changes in the cell cycle-related mRNAs in each group for the short-term culture. The mRNA expressions at day 1 and 5 after seeding in soft agar. (**a**) The expression of Ki-67 mRNA, (**b**) The expression of cyclin B mRNA, (**c**) The expression of CDK1 mRNA, (**d**) The expression of MCM7 mRNA. Bars represent means ± standard error of the mean. n = 3 samples. *P < 0.05, unpaired two-tailed *t*-test. The lower limit of detection (LLOD) is set as the mean expression of MRC-5 at day 5 ± 3.3 × SD. CDK1, cyclin-dependent kinase 1; MCM7, minichromosome maintenance protein 7.

To confirm the requirements, the changes in mRNA levels of Ki-67, cyclin B, CDK1, and MCM7 for short-term culture, i.e., the day after seeding (day 1) and day 5, were determined by PCR (Fig. 2a–d).

In the MRC-5 cells alone group, the expressions of Ki-67, cyclin B, and CDK1 mRNA decreased to approximately 10% by day 5, although that of MCM7 dropped only to 60%. In the two HeLa-spiked groups, the expressions of these markers were higher than that in the MRC-5 alone group due to the proliferation of the HeLa cells.

The criterion of tumorigenicity was defined as the expression above the LLOD, which was calculated as the mean expression of MRC-5 ± 3.3 standard deviations (SD) on the day 5 of culture. LLOD was previously used as a criterion by Kusakawa et al.^5^, who evaluated tumorigenicity using a fluorescent DNA stain in the soft agar colony formation assay.

On the day 5 of culture, Ki-67, cyclin B, and CDK1 levels in the MRC-5 group were very low, and those in the 0.01% and 0.1% HeLa groups were high and exceeded the LLOD, indicating that tumorigenic cells could be detected in both groups. Thus, these markers satisfied both of the above requirements (i) and (ii). Conversely, the MCM7 levels in the 0.01% and 0.1% HeLa groups on day 5 were higher than those in the MRC-5 group; however, those in MRC-5 also remained high (decreased only to 60% from days 1 to 5 of culture), resulting in a high LLOD. Thus, tumorigenicity was only detected in the 0.1% HeLa group. Hence, this marker met only requirement (ii) but not (i).

These results suggest that determining the expression levels of Ki-67, cyclin B, and CDK1 mRNA using PCR could help identify tumorigenic cells that can proliferate in non-anchorage conditions. Although MCM7 expression in MRC-5 was persistently higher than that of Ki-67, cyclin B, and CDK1, this result raises the question of whether a long-term cell culture would lower MCM7 expression in MRC-5 sufficiently to satisfy the requirement (i). In addition, it raises the question of whether other mRNAs that play important roles in cell proliferation, such as CDK2 and proliferating cell nuclear antigen (PCNA), would be useful to evaluate tumorigenicity.

For the second experiment, cells were cultured long-term (up to 4 weeks), and RNA was extracted after weeks 1, 2, 3, and 4 from the day of seeding.

### Ki-67, cyclin B, and CDK1 mRNAs are useful markers, but not CDK2, PCNA, and MCM7, to detect 0.01% of HeLa cells

In the short-term culture, the expressions of Ki-67, cyclin B, and CDK1 mRNA in MRC-5 were strikingly decreased enough to be enabled to set the LLOD to detect 0.01% HeLa cells, whereas in MCM7 was not. Therefore, I thought that if the expression of MCM7 mRNA in MRC-5 is lower than that in the short-term culture, the detection of tumorigenic cells spiked in MRC-5 would be more effective.

For the second experiment, cells were cultured for long-term (up to 4 weeks) and RNA was extracted after 1, 2, 3, and 4 weeks from the day of seeding. Furthermore, two more markers—CDK2 and PCNA—were included in the panel. Because the corresponding proteins are expressed during the G1 and S phases and are involved in cell proliferation, these candidates are expected to provide strong evidence of tumorigenic potency.

**Figure 3:**
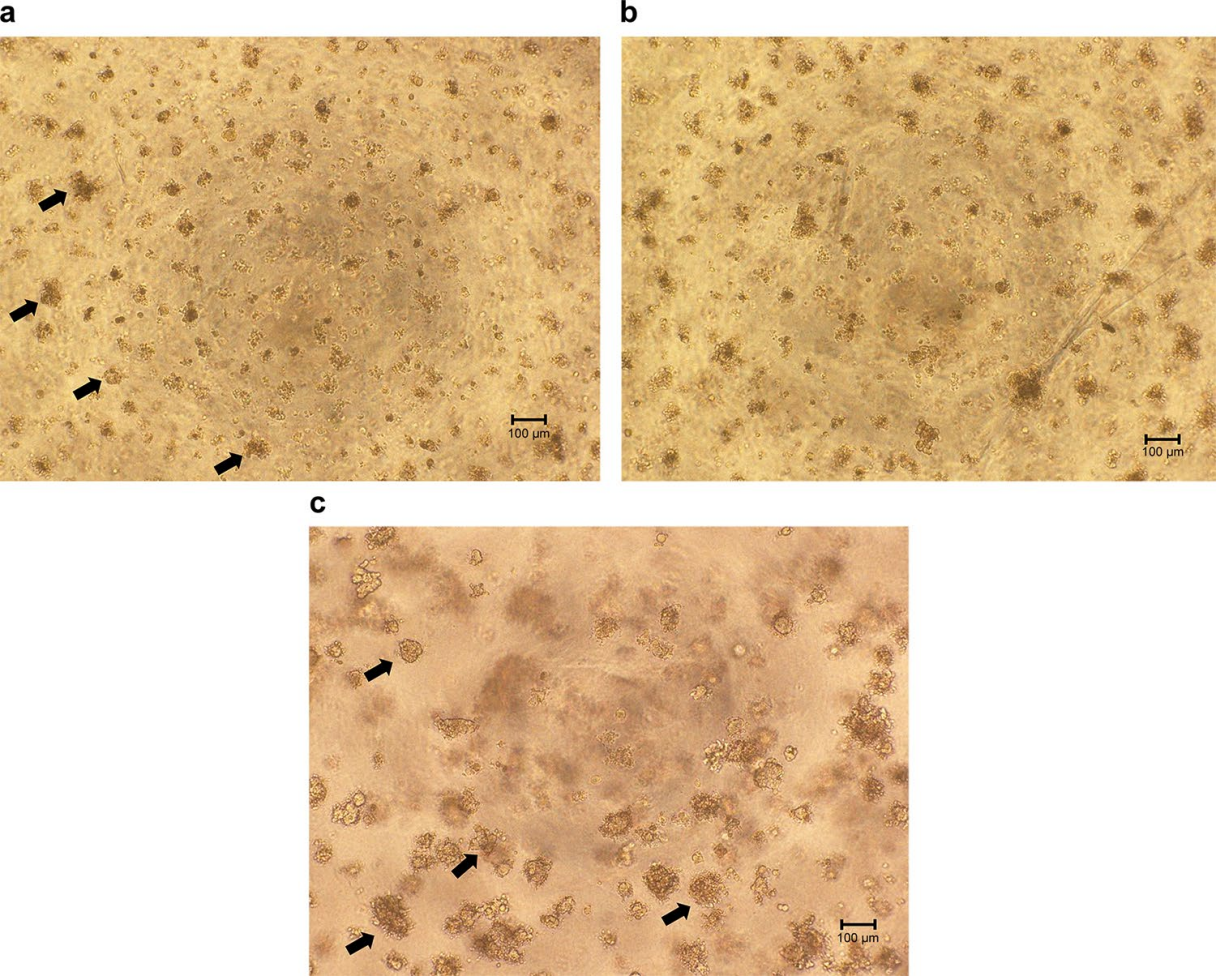
Cells were cultured for 1 week in soft agar during the long-term culture. (**a**) 10^5^ MRC-5 cells/well. Cells aggregated and formed clumps. These are scattered over the entire microscopic field, and some are indicated by arrows. (**b**) 0.1% HeLa cells/well. Colonies of HeLa cells intermingled with clumps of MRC-5 cells but could not be distinguished from each other. (**c**) 10^4^ HeLa cells/well. Many colonies were observed in the soft agar medium; some are indicated by arrows.

**Figure 4:**
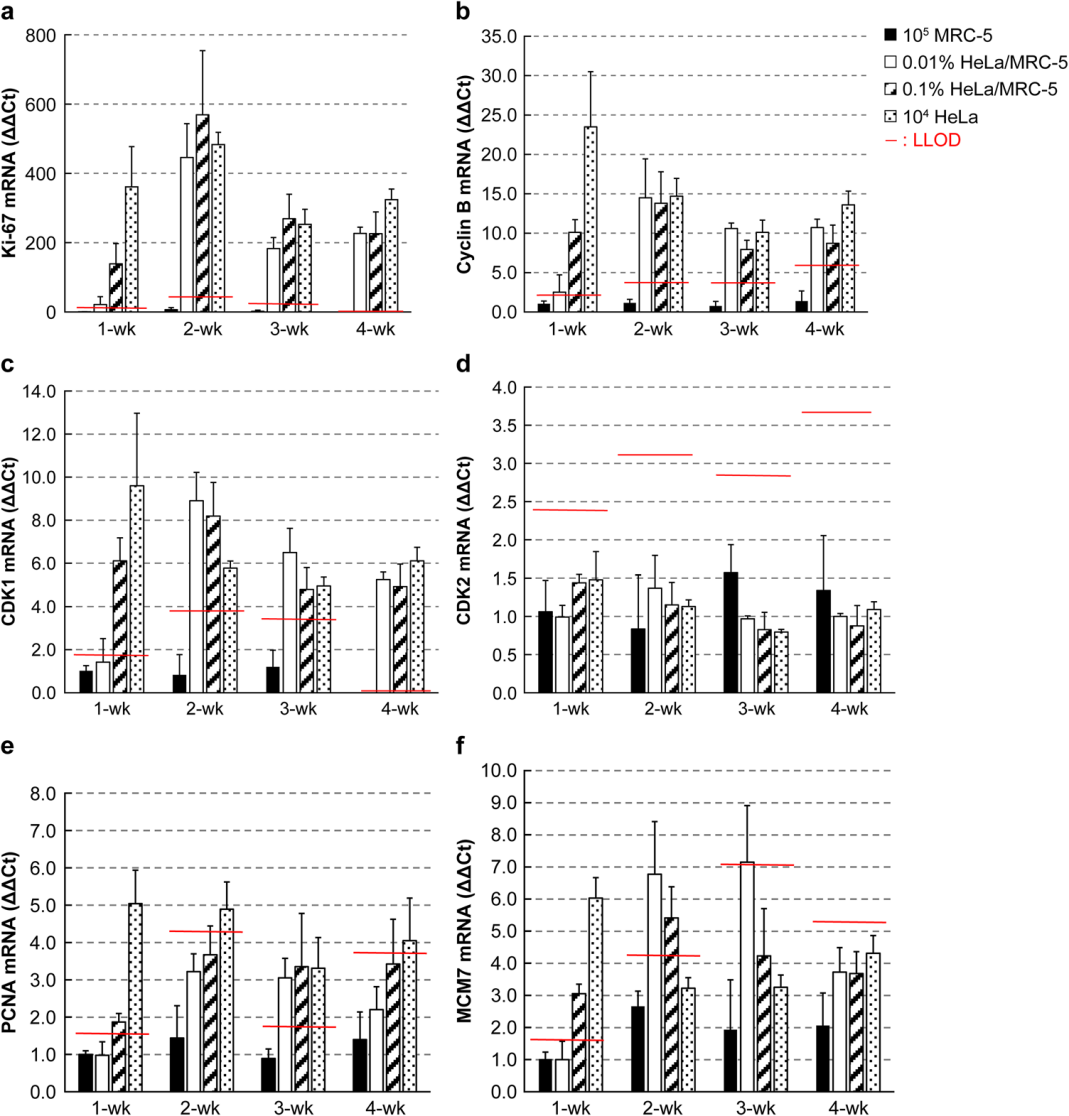
The changes in the cell cycle-related mRNAs in each group for the long-term culture. The mRNA expressions after 1, 2, 3, and 4 weeks of culture in soft agar. (**a**) The expression of Ki-67 mRNA, (**b**) The expression of cyclin B mRNA, (**c**) The expression of CDK1 mRNA, (**d**) The expression of CDK2 mRNA, (**e**) The expression of PCNA mRNA, (**f**) The expression of MCM7 mRNA. Bars represent means ± SD. n = 3 samples. The LLOD is set as the mean expression of MRC-5 at each week ± 3.3 × SD.

In long-term cultures, after 1 week in soft agar, MRC-5 cells aggregated and formed clumps due to high density and not cell proliferation, and these clumps were not distinguishable from the colonies of HeLa cells, which were formed due to cell proliferation (Fig. 3a–c). The mRNA expressions were determined at 1 to 4 weeks from the day of seeding (Fig. 4).

In the MRC-5 alone group, Ki-67, cyclin B, and CDK1 mRNA levels were very low, whereas those in the HeLa-spiked groups were high, and the relative differences between the MRC-5 and HeLa-spiked groups were large. Namely, the maximum relative differences between the MRC-5 and HeLa-spiked groups for Ki-67 was more than 500-fold in the 0.1% HeLa group at 2-week culture, and for cyclin B was more than 20-fold in the HeLa alone group at 1-week culture, respectively. CDK1 showed the maximum relative difference of about 9-fold in the HeLa alone group at 1-week culture, which was smaller than the former two markers.; the expressions of Ki-67 and cyclin B mRNAs in the 0.01% HeLa group exceeded the LLOD in 1-week culture, this is similar to the short-term culture, confirming that these markers could detect 0.01% of tumorigenic cells after a week of agar culture. Moreover, in more than 2-week cultures, the expressions of these markers were consistently above the LLOD in all of the 0.01%, 0.1%, and HeLa alone groups, indicating that tumorigenic cells could be detected. Conversely, CDK1 mRNA of 0.1% HeLa and HeLa alone groups in 1-week culture exceeded the LLOD, but 0.01% HeLa was slightly less than the LLOD. Hence, the resulting tumorigenicity could be detected only in the 0.1% HeLa and HeLa alone groups. 0.01% HeLa was not detected at 1 week in the long-term experiment, although it was detected at 5 days in the short-term experiment. However, in more than 2-week culture, CDK1 expressions were more than the LLOD in all groups, including 0.01% HeLa, and thus tumorigenicity could be detected in all groups.

On the other hand, when CDK2, PCNA, and MCM7 were used as markers, their expression remained high in MRC-5 cells even after 4 weeks of culture, and the relative differences between MRC-5 and HeLa-spiked groups were smaller than those between Ki-67, cyclin B, and CDK1. These results led to higher LLODs and an unstable detection sensitivity. CDK2 mRNA expression in the MRC-5 group was not reduced and remained, resulting in higher LLOD, and consequently no tumorigenicity was detected in 0.01%, 0.1% HeLa, and HeLa alone groups at all culture times. For PCNA expression, the relative differences between MRC-5 and HeLa-spiked groups were smaller than those between Ki-67, cyclin B, and CDK1, peaking at about 5-fold in the 1-week culture of HeLa alone group. In the 1-week culture, PCNA levels exceeded the LLOD in the 0.1% HeLa and HeLa alone group, and the tumorigenic cells were detected in both the groups, but in the 2-week, tumorigenic cells were detected only in the HeLa alone, but not in the 0.01% and 0.1% HeLa group. At 3 weeks, the PCNA expressions in the 0.01%, 0.1% HeLa, and HeLa alone group were more than the LLOD, detecting the tumorigenicities in all groups, but only in the HeLa alone group and not in the other groups at 4 weeks. As for MCM7 expression, the relative differences between the MRC-5 and HeLa-spiked groups were also smaller than that of Ki-67, cyclin B, and CDK1, with the maximum of about 7-fold in 3-week culture of 0.01% HeLa group. MCM7 expression exceeded the LLOD in the 0.1% HeLa and HeLa alone groups in 1-week culture, and tumorigenic cells were detected in both the groups, whereas in 2-week cultures, tumorigenic cells were detected in the 0.01% and 0.1% HeLa groups only, but not in the HeLa alone group. After 3-week culture, only in the 0.01% HeLa group, MCM7 expression exceeded the LLOD and tumorigenic cells were detected, but after 4 weeks, no tumorigenic cells were detected in all the groups.

Therefore, CDK2, PCNA, and MCM7 would not be appropriate for tumorigenicity detection.

### Soft agar colony assay using PCR can detect CSC markers

**Figure 5:**
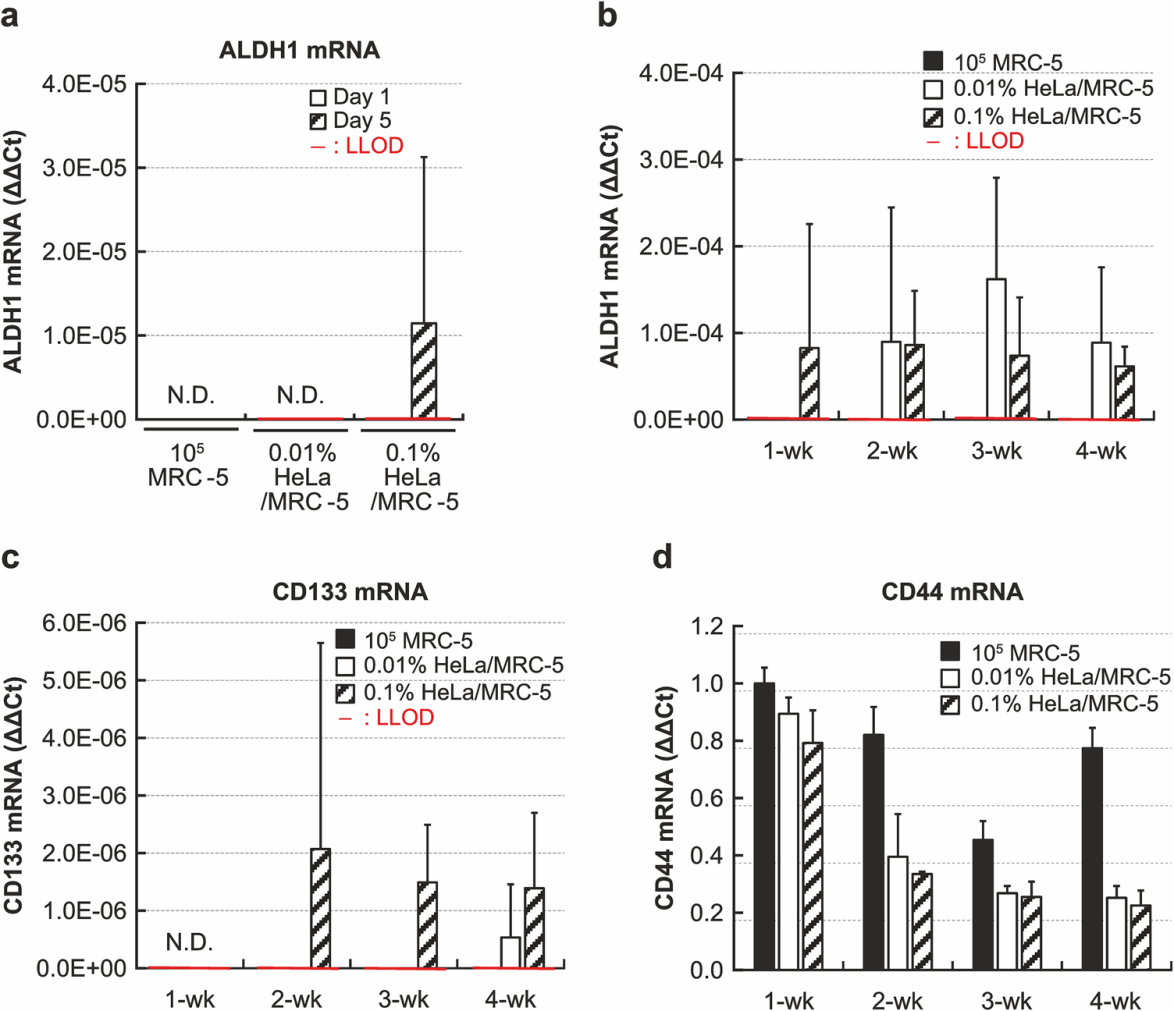
The changes in the cancer stem cell markers in the short and long-term culture. The mRNA expressions in soft agar of the short- and long-term culture. (**a**) The expression of ALDH1 mRNA in the short-term culture, (**b**) The expression of ALDH1 mRNA in the long-term culture, (**c**) The expression of CD133 mRNA in the long-term culture, (**d**) The expression of CD44 mRNA in the long-term culture. The LLOD of CD44 mRNA was not calculated because its expression in the MRC-5 alone group was much higher than the groups containing HeLa cells. Bars represent means ± SD. n = 3 samples. The LLOD is set as mean expression of MRC-5 at each week ± 3.3 × SD. ALDH1, aldehyde dehydrogenase 1.

The mRNAs of aldehyde dehydrogenase 1 (ALDH1), CD133, and CD44 were measured as makers of CSCs (Fig. 5). ALDH1 contributes to cancer cell survival by participating in cell proliferation, drug-resistance mechanisms, anti-oxidant pathways, and metastasis^15^. CD133 is involved in glucose uptake and localizes to the cellular surface^16^. In the short-term culture, ALDH1 could be detected in 0.1% of HeLa cells of the 5-day culture but not in the MRC-5 and 0.01% HeLa groups (Fig. 5a). In the long-term cultures, ALDH1 was detected in 0.1% HeLa in 1-week culture, but not in the MRC-5 and the 0.01% HeLa groups (Fig. 5b), and this result was similar to that in the short-term experiment. Furthermore, this marker was detected in 0.01% of HeLa cells after 2 weeks of culture and continued to be detected in more than 3 weeks of culture. On the other hand, CD133 was not detected in the short-term culture (data not shown), and was detectable in 0.1% of HeLa cells after 2 weeks and 0.01% of HeLa cells after 4 weeks of culture (Fig. 5c), whereas this mRNA could not be detected in MRC-5 cells alone.

CD44 is also a well-known CSC marker, which localizes to the cell surface and is involved in cell division, migration, and adhesion^16^. However, CD44 mRNA was detected in MRC-5 alone group also, besides the HeLa-spiked groups (Fig. 5d). This result suggests that appropriate markers for CSC should be considered.

## Discussion

The soft agar colony formation assay is very useful for evaluating the tumorigenicity of CTPs^4,5^, and can detect as low as 0.00001% HeLa cells spiked in mesenchymal stem cells. However, it takes 20–30 days to complete the assay. I propose an approach that can detect tumorigenic cells much faster by measuring the level of certain mRNAs. These are proliferation markers which can help identify the presence of cells in the soft agar that can proliferate without scaffolds. mRNA expression is strictly regulated according to time and the specific stage of the cell, such as cell growth, differentiation, etc., ensuring the time-efficiency of this method.

In this study, by measuring specific mRNAs from cells grown in soft agar, I could detect 0.01% HeLa cells spiked into MRC-5 cells after 5 days of culture. Furthermore, this assay could detect CSC markers from 0.01% HeLa cells after 2 weeks of culture. Therefore, it could be a useful tool for screening the tumorigenic risk of the ingredients or candidates for CTPs in the early stages of their development.

The mRNAs examined in this study, namely Ki-67, cyclin B, CDK1, CDK2, PCNA, and MCM7, were selected because their proteins play essential roles in DNA replication or mitosis. In the long-term culture, the results showed that the expressions of Ki-67, cyclin B, and CDK1 in the HeLa-spiked groups were much higher; the maximum relative differences of the markers being approximately 9–500-fold higher than that in MRC-5. These mRNAs exceeded the LLODs calculated from that of the MRC-5 group in each culture period, thereby enabling to detect the tumorigenicity of 0.01% and 0.1% HeLa cells. When CDK1 was used as a marker, 0.01% HeLa was detected after 5 days in the short-term culture experiment, but in the long-term culture, it was slightly less than the LLOD at 1 week and exceeded the LLOD at 2-week, becoming detectable.

As a reason for the low sensitivity of CDK1, it was considered that the relative differences in CDK1 expression between the MRC-5 alone and HeLa-added groups were smaller (about 9-fold at max) than that of Ki-67 and cyclin B (about 500-fold and about 20-fold at max, respectively). For CDK2, PCNA, and MCM7, the relative differences in expression between the MRC-5 alone and HeLa-spiked groups were smaller than those for the former three markers, and the largest relative difference was about 7-fold of MCM7 in the 0.01% HeLa group at 3-week culture. As a result, higher LLODs resulted in a lower detection sensitivity, and HeLa cells could not be detected in some groups or culture times. Therefore, Ki-67, cyclin B, and CDK1 are useful markers to assess tumorigenicity, whereas CDK2, PCNA, and MCM7 are not.

Ki-67 expresses during all phases of the cell cycle, except G0^17,18^. Therefore, this protein is a prominent cell proliferation marker in pathology and the Ki-67 labeling index is used in the diagnosis and prognosis of cancers, such as breast cancer^19^, meningiomas^20^, and in the WHO classification of tumors of the digestive system^21^. The Ki-67 protein localizes to the surface of chromosomes^22,23^ and acts as a surfactant to prevent chromosomes coalescing during mitosis^24^. On the other hand, CDK1 and cyclin B express in the M phase and form their own complexes, contributing to spindle assembly^25^. These three markers decreased in MRC-5 cells to approximately 10% after 5 days of culture in soft agar. These results increase the confidence in this method to evaluate the tumorigenicity of CTPs.

CDK2, PCNA, and MCM7 are also known cell proliferation factors and these proteins express in the G1 and S phase. CDK2 actives the E2F transcription factor indirectly by inhibiting the Rb gene, followed by activation of transcription factors related to cell proliferation^26^. PCNA is used as a cell proliferation marker in pathological diagnostics and helps DNA polymerases bind to DNA^27^. MCM7, a component of the MCM2-7 complex that acts as a DNA helicase in the replication fork^28^, is expected to be a tumor progression marker^29,30^. However, in this study, these mRNAs were detected in MRC-5 cells even after 4 weeks of culture in soft agar, probably due to differences in mRNA degradation rate.

Several studies report that CSCs originated from iPS cells, although they use artificially conditioned medium^7,31,32^. Another study discussed the risk of CSCs accidentally emerging in CTPs or their raw materials^33^. Using the proposed method, I could detect the CSC markers ALDH1 and CD133 after 2 and 4 weeks, respectively, in 0.01% HeLa cells, while they were not detected in MRC-5 cells. ALDH1, CD44, and CD133 are expressed in CSCs in several cancers, including breast, gastric, brain, lung, liver, colon, and pancreas^6^, which can be candidate organs for cytotherapies. However, the CSC markers are still not clearly defined. CD44 and CD133 are expressed in normal stem cells^16^, and in this study, CD44 mRNA was actually detected in MRC-5 fibroblasts. Therefore, while suitable markers for the detection of various CSCs need to be investigated, the PCR method might be a useful tool to characterize colonies in soft agar.

In cancer therapy, CSCs have been intensely researched in recent years, and CSC markers, such as CD44 and CD133, have been shown to be enhanced in three-dimensional spheroid cultures than in two-dimensional monolayer cultures, since these cultures mimic the in vivo microenvironment^34–37^. Separately, culture on soft agar has also been widely adopted because colony-forming ability is an indicator of cancer metastatic potential. In the present study, since mRNA could be detected even from a small number of HeLa cells (0.01%), the identification and characterization of a small number of CSCs in cancer tissues collected from patients is possible. Therefore, this method may also be a useful tool in CSC research to simultaneously examine soft agar colony-forming ability and PCR analysis of CSC markers.

This study had some limitations. First, the detection sensitivity was not sufficient, for example, 2 × 10^8^ cells/person are required for the treatment of ischemic heart disease^38^. Therefore, although one 96-well plate was used in this study, this method can be scaled up by using more 96-well plates, for example, to culture a larger number of cells. Second, this method was not tested on other tumorigenic cells contaminating actual CTPs, their intermediates, or raw materials. Third, the mRNA level determined by PCR does not constitute absolute evidence of tumorigenicity because the number of cells generated from tumorigenic cells is not directly observed; therefore, a combination of some mRNAs would be needed to evaluate the tumorigenicity, because the PCR amplification efficiency might be affected if there was a mutation in the DNA coding for these mRNAs. Finally, appropriate markers of CSCs should be investigated because some markers, such as CD44, are often expressed in normal stem cells.

In conclusion, by using PCR to measure mRNAs from colonies in soft agar, I could detect 0.01% HeLa cells spiked into MRC-5 cells after 5-day of culture, and CSC markers after 2 weeks of culture. Thus, this study suggests that mRNA measurement in the soft agar colony formation assay could be a useful tool for screening the tumorigenic risk in the short-term and characterizing the colonies representing the ingredients or candidates for CTPs during their early developmental stages.

## Methods

### Cells

MRC-5, a non-tumorigenic normal cell line, and HeLa cells, a tumorigenic cancer cell line, were used in this study as a negative and positive control, respectively. Both these lines were purchased from the JCRB Cell Bank at the National Institutes of Biomedical Innovation (Osaka, Japan). These cells were maintained in Dulbecco's Modified Eagle Medium–high glucose (DMEM-high glucose; Thermo Fisher Scientific, MA, USA) supplemented with 10% fetal bovine serum (FBS; Thermo Fisher Scientific, MA, USA), 100 U/mL penicillin, and 100 µg/mL streptomycin (Thermo Fisher Scientific, MA, USA). Cells were cultured in an incubator set at 5% CO_2_ and 37 °C.

### Soft agar colony formation assay

The soft agar colony formation assay kit (Cell Biolabs, CA, USA) was used to detect the tumorigenic potency of the two cell lines according to the manufacturer’s instructions. First, to prevent the cells from adhering to the bottom of the cell culture plate, the bottom agar was prepared as follows: 50 µL of 0.6% agar in DMEM, containing 10% FBS, was pre-warmed to 37 °C, transferred into clear flat-bottomed 96-well plates (PerkinElmer Japan, Tokyo, Japan), and chilled at 4 °C to solidify the bottom agar. Cells were harvested and dissociated into single-cell suspensions using a 0.25% trypsin–EDTA solution (Gibco, MA, USA). The cells were adjusted to the targeted cell density by diluting with DMEM and mixed into 0.6% agar at a ratio of 1:2, respectively, to obtain a final concentration of 0.4% agar containing cells. For each well, 75 µL of the 0.4% agar containing cells was layered onto the 0.6% bottom agar in the 96-well plates and chilled at 4 °C for 20 min to solidify the cell agar. After solidification, 100 µL per well of complete DMEM (with supplements) was added to the cell agar and the cells were cultured at 37 °C in 5% CO_2_. The medium was changed every 5–6 days until the end of the culture term described below. The assays were conducted in triplicates.

### The term of cell culture in the soft agar

In this study, two types of experiments—short and long-term culture—were conducted. For the short-term experiment, to observe the changes in expression of mRNAs involved in the cell cycle, the cells were cultured up to 5 days and mRNA samples were extracted on the day after seeding (day 1) and day 5. Four groups were set up as follows: blank (absent cells), 10^5^ MRC-5 cells alone/well, 10 HeLa cells spiked in 10^5^ MRC-5 cells (0.01% HeLa)/well, and 100 HeLa cells spiked in 10^5^ MRC-5 cells (0.1% HeLa)/well.

For the long-term experiments, the cells were cultured for 4 weeks and mRNA samples were extracted on weeks 1, 2, 3, and 4. Five groups were set up as follows: the four groups used in the short-term experiment plus 10^4^ HeLa cells alone/well.

### RNA extraction from soft agar

RNA was extracted from soft agar using acetic acid, as reported by Mio et al.^39^, who demonstrated that lowering the pH by adding sodium acetate could efficiently extract RNA by neutralizing the electrostatic repulsion between the nucleic acid and silica membrane. At the end of the culture, the DMEM on the cell agar was discarded, the agar dissolved in a total of 525 µL per well of RLT buffer (QIAGEN), and the agar solutions lysed using the QIA shredder (QIAGEN). Then, 5 µL acetic acid (Fuji Film Wako, Osaka, Japan) and 366 µL ethanol (Fuji Film Wako, Osaka, Japan) were added to each agar lysate and mixed vigorously. In the following steps, the RNeasy® Mini Kit (QIAGEN) with DNase I treatment (QAIGEN) was used according to the manufacturer’s instructions.

### Quantitative real-time PCR (qPCR)

**Figure 6:**
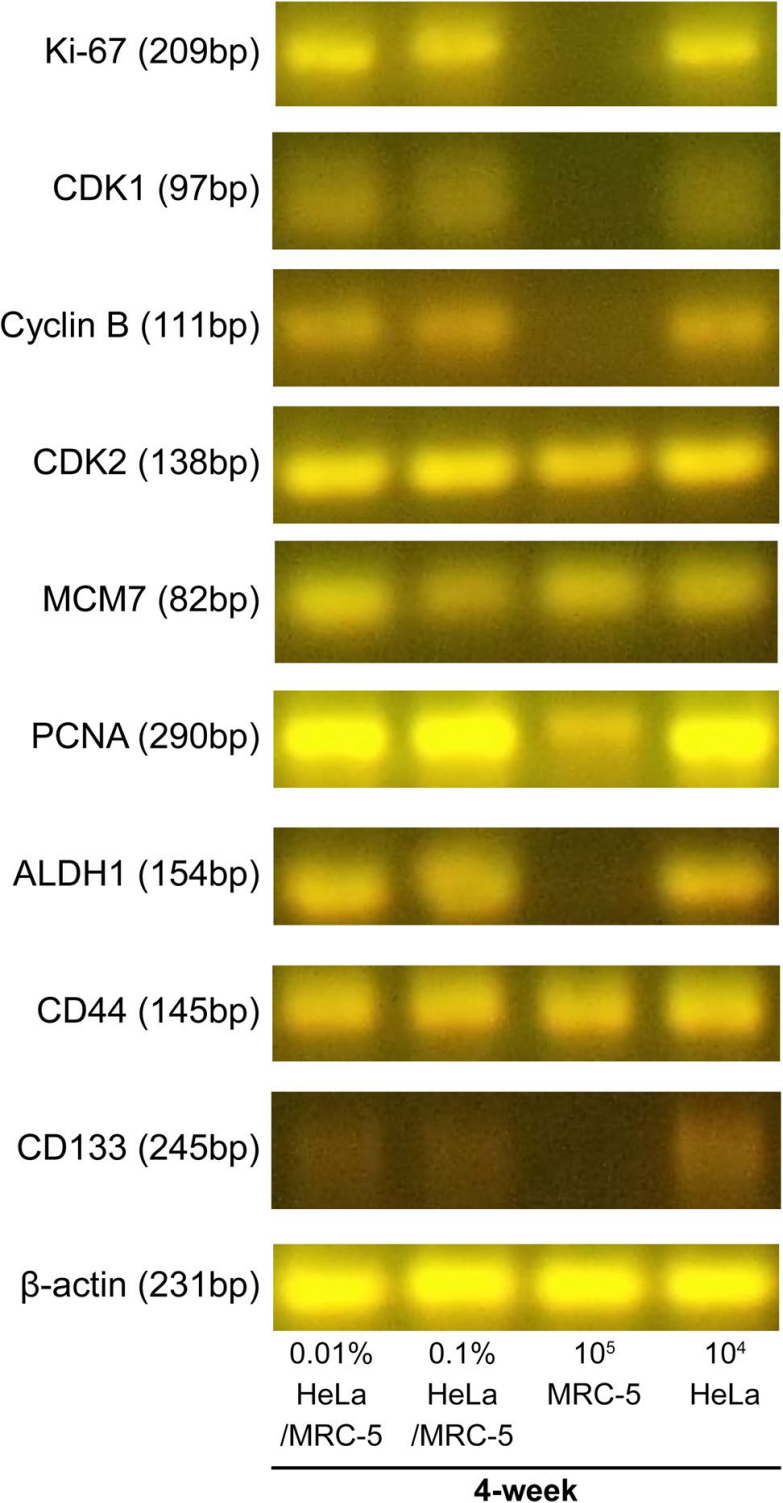
Electrophoresis of PCR products from 4-week cultured cells in soft agar for the long-term culture. PCR products of cell cycle-related mRNAs, CSC markers, and β-actin mRNA. The PCR products of the 0.01% HeLa, 0.1% HeLa, and 10^4^ HeLa alone group were observed at the expected size. On the other hand, in the 10^5^ MRC-5 alone group, Ki-67, CDK1, cyclin B, ALDH1, and CD133 mRNA were not detected at the expected size. CSC, cancer stem cell.

In this study, the mRNA markers for cell proliferation were Ki-67, cyclin B, CDK1, CDK2, PCNA, and MCM7, whereas those for CSCs were ALDH1, CD44, and CD133. For the qPCR, 3 µL of RNA sample was used to synthesize cDNA at a 30 µL scale using the Superscript VILO cDNA synthesis kit (Thermo Fisher Scientific, MA, USA) according to manufacturer’s instructions. The reaction conditions were as follows: 25 °C for 10 min, 42 °C for 60 min, and 85 °C for 5 min, using the GeneAmp PCR System (Thermo Fisher Scientific, MA, USA). The qPCR reaction was performed using the Fast SYBR Green PCR master Mix (Thermo Fisher Scientific, MA, USA) in a 7500 Real-Time PCR System (Thermo Fisher Scientific, MA, USA). The cycling conditions were: one cycle of 20 s at 95 °C, 40 cycles of 3 s at 95 °C and 30 s at 60 °C. β-actin mRNA was used as a housekeeping gene to normalize the expression of the markers. The primer sequences are shown in Table 1. The size of PCR products was confirmed by gel electrophoresis (Fig. 6 and Supplementary Information) at 100 V for 25 min in 4% agarose 21 (NIPPON GENE, Tokyo, Japan) containing GelStar Nucleic Acid Gel Stain (LONZA, ME, USA) dissolved in TAE buffer (NIPPON GENE, Tokyo, Japan).

**Table 1:** Information for qPCR primers.

Human genes	
Gene	Sequence
*Ki-67*	F: 5′-ACGCCTGGTTACTATCAAAAGG-3′
	R: 5′-CAGACCCATTTACTTGTGTTGGA-3′
*CDK1*	F: 5′-GTGCTTATGCAGGATTCCAGGT-3′
	R: 5′-CCATGTACTGACCAGGAGGGA-3′
*CDK2*	F: 5′-TGGCGCTTCATGGAGAACTT-3′
	R: 5′-ACCCTCAGTCTCAGTGTCCA-3′
*Cyclin B*	F: 5′-AATAAGGCGAAGATCAACATGGC-3′
	R: 5′-TTTGTTACCAATGTCCCCAAGAG-3′
*PCNA*	F: 5′-TGTTGGAGGCACTCAAGGAC-3′
	R: 5′-AACTTTCTCCTGGTTTGGTGC-3′
*MCM7*	F: 5′-CCTACCAGCCGATCCAGTCT-3′
	R: 5′-CCTCCTGAGCGGTTGGTTT-3′
*CD44*	F: 5′-ATCATCTTGGCATCCCTCTTG-3′
	R: 5′-TGAGTCCACTTGGCTTTCTG-3′
*CD133*	F: 5′-CAGAGTACAACGCCAAACCA-3′
	R: 5′-AAATCACGATGAGGGTCAGC-3′
*ALDH1A*	F: 5′-TGTTAGCTGATGCCGACTTG-3′
	R: 5′-TTCTTAGCCCGCTCAACACT-3′
*β-Actin*	F: 5′-GCCGCCAGCTCACCA-3′
	R: 5′-GCTCGATGGGGTACTTCAGG-3′

### Statistical analysis

The mRNA expressions on the day after seeding (day 1) and day 5 (short-term culture) are presented as mean ± standard error of the mean. Statistical analysis was performed using Prism 5 (Graph Pad, ver. 5.0.4). Statistical significance for the expression levels was determined by an unpaired two-tailed* t*-test. *P* < 0.05 denotes statistical significance.

## Supplementary Information

Supplementary Information.

## Data Availability

The datasets used and/or analyzed during the current study available from the corresponding author on reasonable request.
