# Supplementary material for: The evaluation of tumorigenicity and characterization of colonies in a soft agar colony formation assay using polymerase chain reaction
Source: Sci Rep. 2023 Apr 3;13:5405. doi: 10.1038/s41598-023-32442-6 (PMC10070612; doi:10.1038/s41598-023-32442-6)
Supplement: Supplementary file 1 — Supplementary Information. [file 41598_2023_32442_MOESM1_ESM.pptx]

## Slide 1
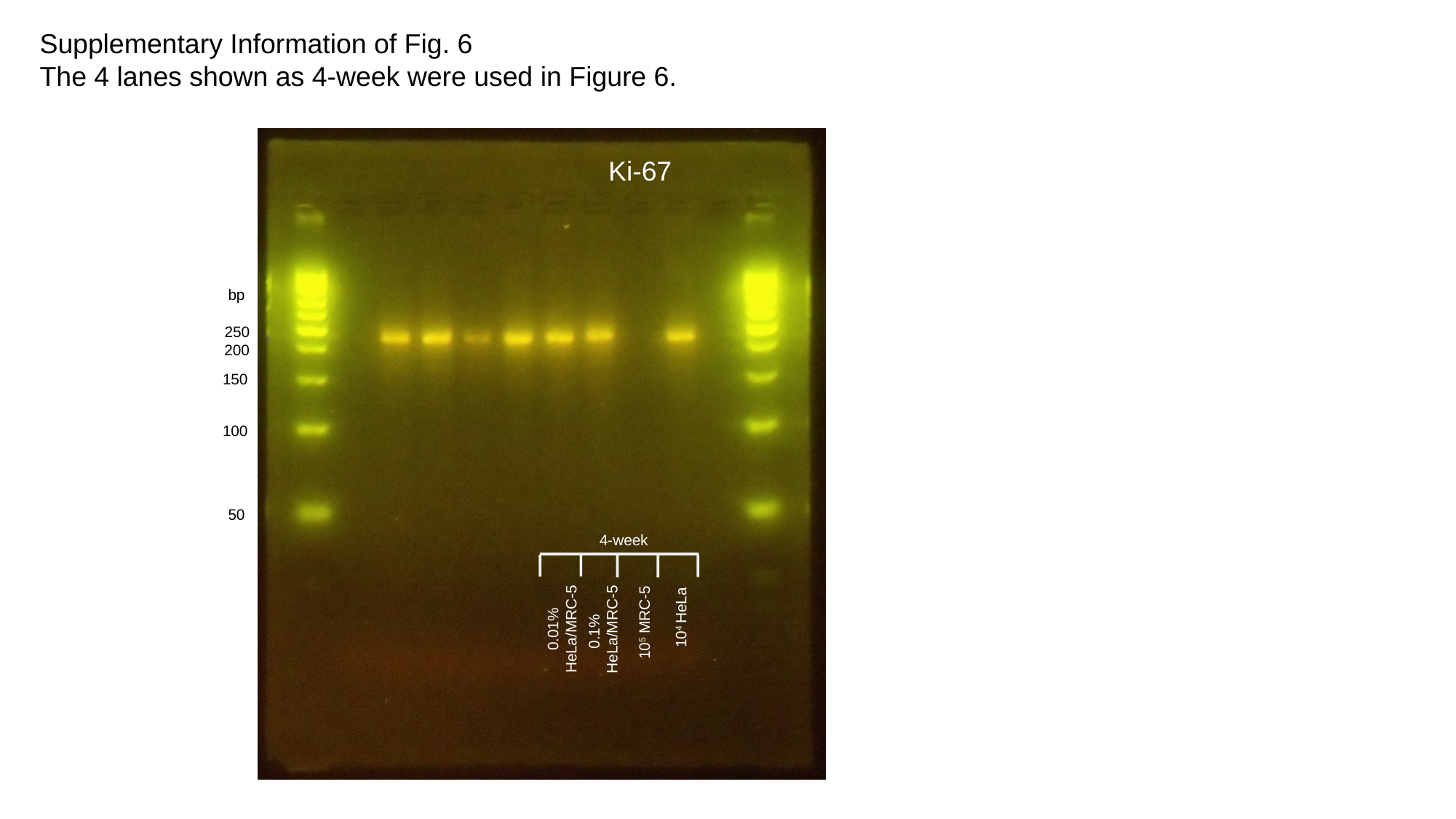

Supplementary Information of Fig. 6
The 4 lanes shown as 4-week were used in Figure 6.
Ki-67
bp
200
150
100
50
250
4-week
104 HeLa
0.01% HeLa/MRC-5
105 MRC-5
0.1%
 HeLa/MRC-5

## Slide 2
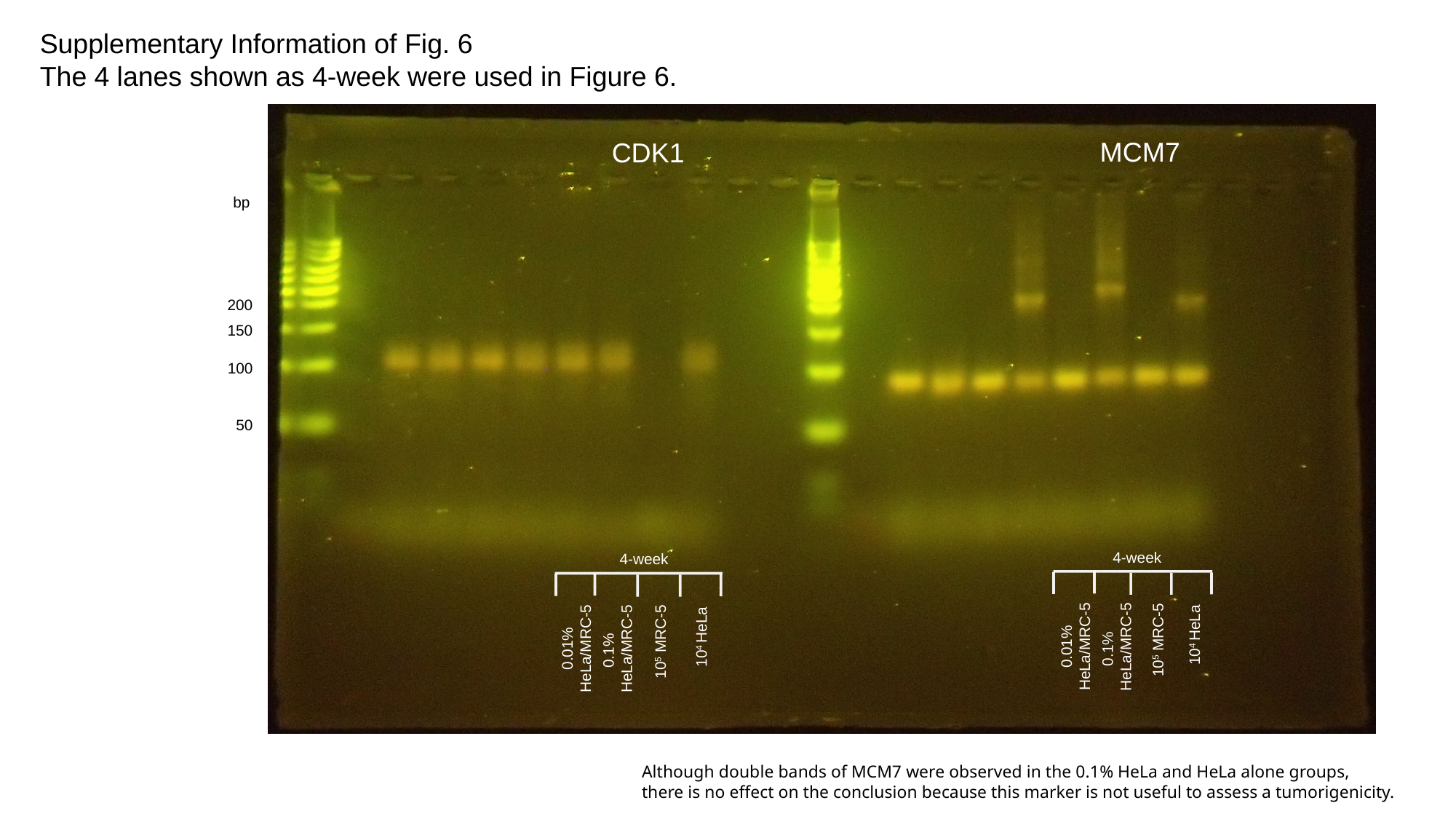

Supplementary Information of Fig. 6
The 4 lanes shown as 4-week were used in Figure 6.
MCM7
CDK1
bp
200
150
100
50
4-week
104 HeLa
0.01% HeLa/MRC-5
105 MRC-5
0.1%
 HeLa/MRC-5
4-week
104 HeLa
0.01% HeLa/MRC-5
105 MRC-5
0.1%
 HeLa/MRC-5
Although double bands of MCM7 were observed in the 0.1% HeLa and HeLa alone groups,
there is no effect on the conclusion because this marker is not useful to assess a tumorigenicity.

## Slide 3
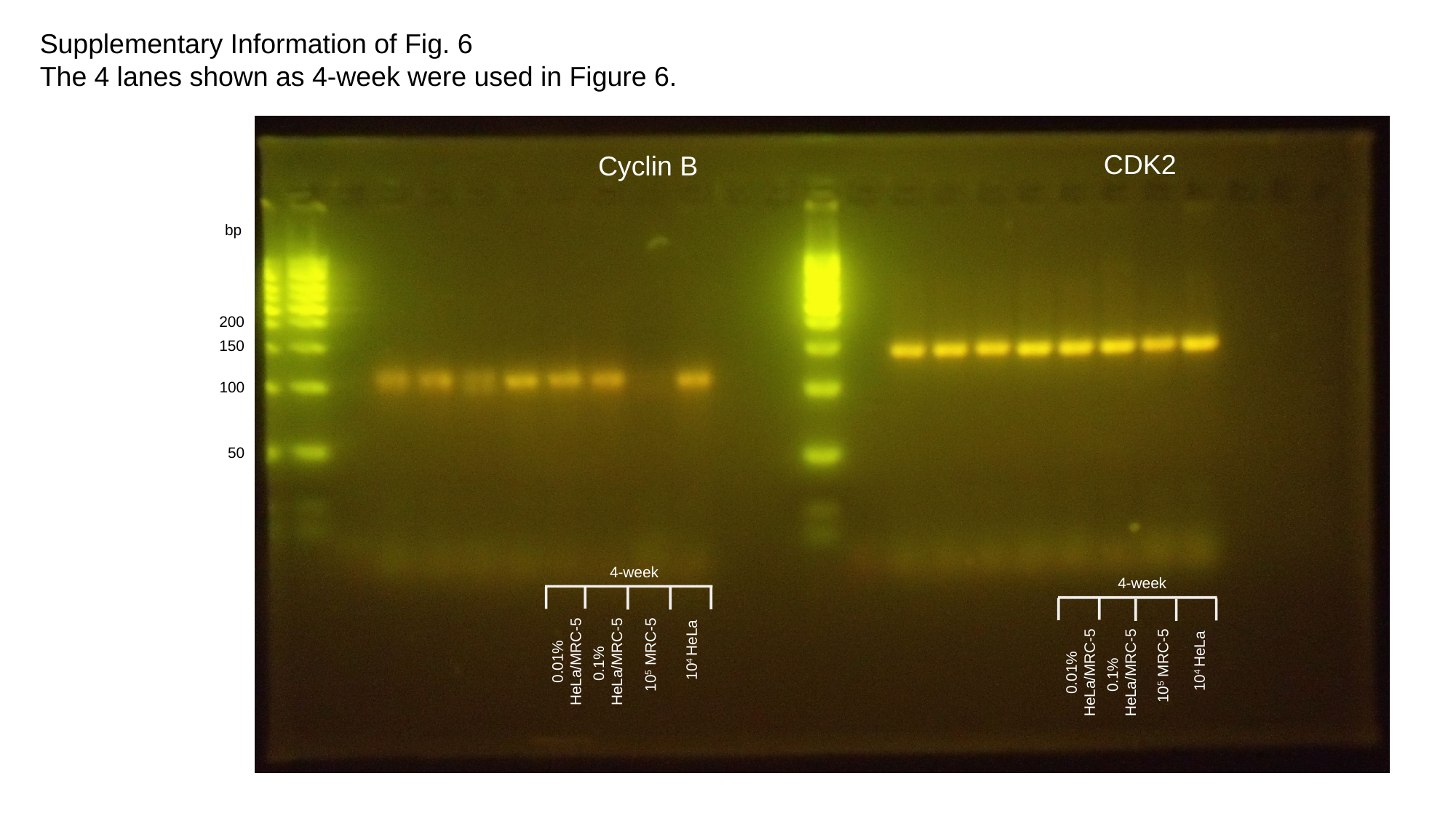

Supplementary Information of Fig. 6
The 4 lanes shown as 4-week were used in Figure 6.
CDK2
Cyclin B
bp
200
150
100
50
4-week
104 HeLa
0.01% HeLa/MRC-5
105 MRC-5
0.1%
 HeLa/MRC-5
4-week
104 HeLa
0.01% HeLa/MRC-5
105 MRC-5
0.1%
 HeLa/MRC-5

## Slide 4
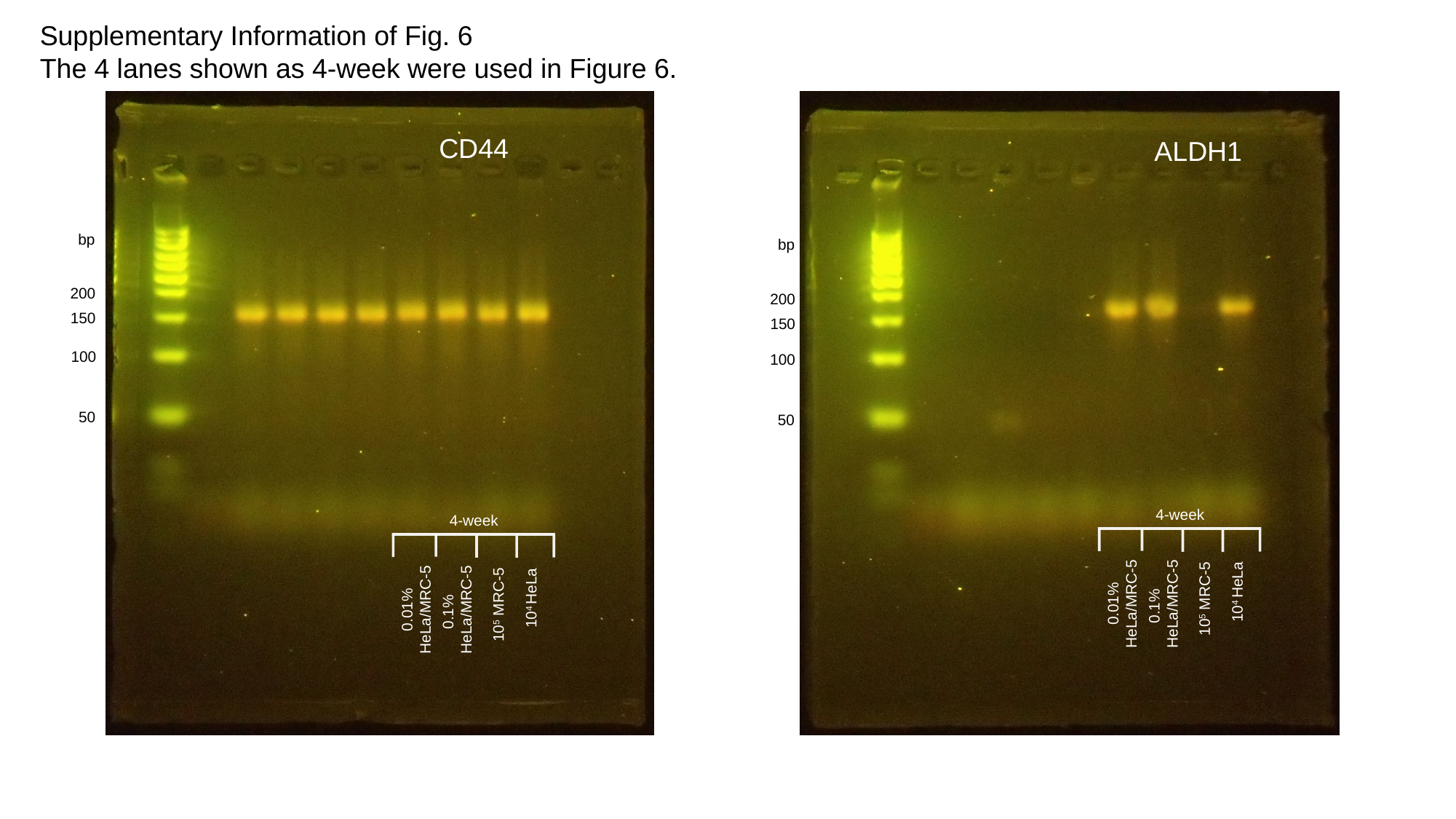

Supplementary Information of Fig. 6
The 4 lanes shown as 4-week were used in Figure 6.
CD44
bp
200
150
100
50
4-week
104 HeLa
0.01% HeLa/MRC-5
0.1%
 HeLa/MRC-5
105 MRC-5
ALDH1
bp
200
150
100
50
4-week
104 HeLa
0.01% HeLa/MRC-5
0.1%
 HeLa/MRC-5
105 MRC-5

## Slide 5
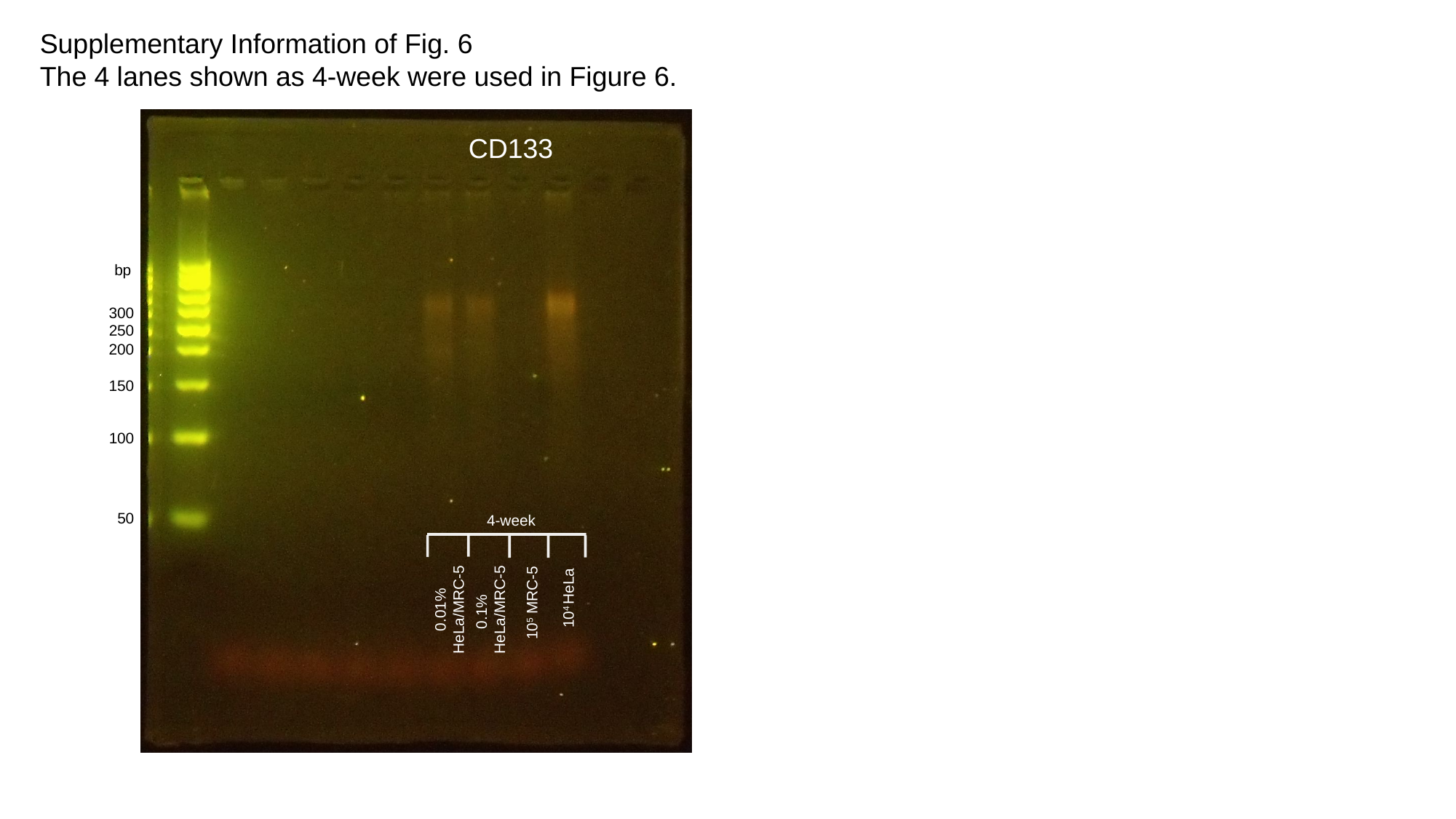

Supplementary Information of Fig. 6
The 4 lanes shown as 4-week were used in Figure 6.
CD133
bp
200
150
100
50
300
250
4-week
104 HeLa
0.01% HeLa/MRC-5
105 MRC-5
0.1%
 HeLa/MRC-5

## Slide 6
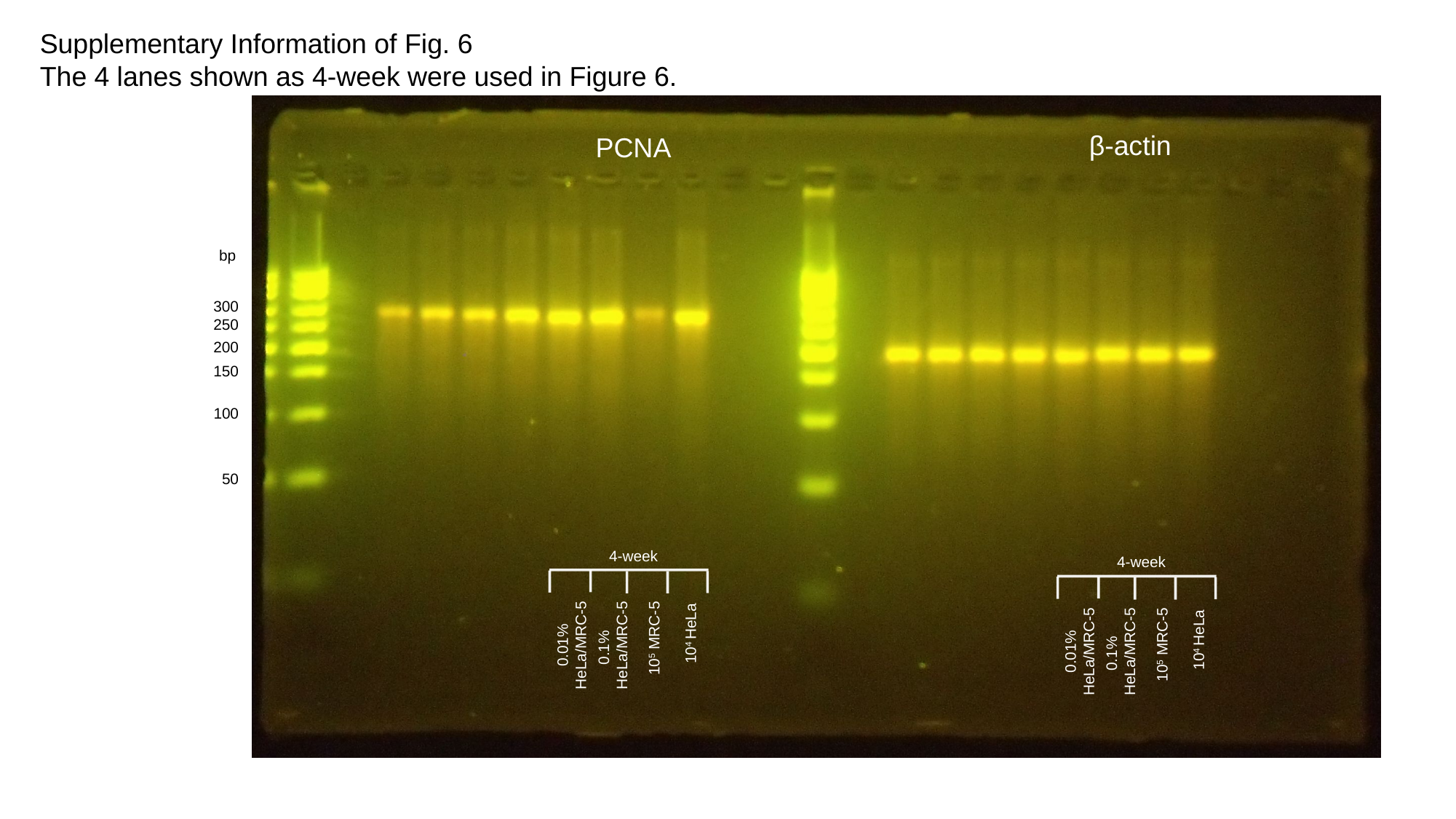

Supplementary Information of Fig. 6
The 4 lanes shown as 4-week were used in Figure 6.
β-actin
PCNA
bp
200
150
100
50
300
250
4-week
104 HeLa
0.01% HeLa/MRC-5
105 MRC-5
0.1%
 HeLa/MRC-5
4-week
104 HeLa
0.01% HeLa/MRC-5
105 MRC-5
0.1%
 HeLa/MRC-5
